# The efficacy of lumbar erector spinae plane block for postoperative analgesia management in patients undergoing lumbar unilateral bi-portal endoscopic surgery: a prospective randomized controlled trial

**DOI:** 10.1186/s12871-024-02601-x

**Published:** 2024-07-01

**Authors:** Dan Zhao, Hongkun Wang, Xin Liu, Zhenfeng Gao, Chao Sun, Quanyi Zhang

**Affiliations:** 1 Department of Anesthesiology, Binzhou Medical University Hospital, No. 661 Huanghe 2nd Road, Binzhou, Shandong Province 256603 China; 2 Department of Rehabilitation Medicine, Binzhou Medical University Hospital, Binzhou, Shandong Province 256603 China; 3 Department of Spinal Surgery, Binzhou Medical University Hospital, Binzhou, Shandong Province 256603 China; 4 Department of Anesthesiology and Reanimation, Binzhou Medical University Hospital, Binzhou, Shandong Province 256603 China

**Keywords:** Lumbar, Erector spinae plane block, Unilateral bi-portal endoscopic, Quality of recovery, Postoperative analgesia management

## Abstract

**Background:**

The efficacy and reliability of erector spinae plane block (ESPB) in posterior open lumbar spine surgery has been demonstrated; however, few randomized controlled trials of lumbar ESPB (L-ESPB) in lumbar unilateral bi-portal endoscopic (UBE) surgery have been reported.

**Methods:**

A total of 120 patients, aged 18 to 65 (who underwent elective lumbar UBE surgery under general anesthesia and exhibited an American Society of Anesthesiologists physical status of I to III) were randomly assigned in a 1:1 ratio to the ESPB group and the Control group. Ultrasound(US)-guided unilateral single-shot 0.25% ropivacaine L-ESPB was performed in the ESPB group, but not in the control group. Postoperative analgesic strategy for all patients: patient controlled intravenous analgesia (PCIA, diluted and dosed with fentanyl alone) was initiated immediately after surgery combined with oral compound codeine phosphate and ibuprofen sustained release tablets (1 tablet containing ibuprofen 200 mg and codeine 13 mg, 1 tablet/q12h) commenced 6 h postoperatively. We collected and compared patient-centred correlates intraoperatively and 48 h postoperatively. The primary outcomes were intraoperative and postoperative opioid consumption and postoperative quality of recovery-15 (QoR-15) scores.

**Results:**

Compared to the control group (*n* = 56), the ESPB group (*n* = 58) significantly reduced intraoperative remifentanil consumption (estimated median difference − 280 mcg, 95% confidence interval [CI] − 360 to − 200, *p* < 0.001, power = 100%); significantly reduced fentanyl consumption at 24 h postoperatively (estimated median difference − 80mcg, 95%[CI] − 128 to − 32, *p* = 0.001, power = 90%); and significantly enhanced the QoR-15 score at 24 h postoperatively (estimated median difference 11, 95%[CI] 8 to 14, *p* < 0.001, power = 100%). Compared to the control group, the ESPB group enhanced the resting numeric rating scale (NRS) score up to 8 h postoperatively, and the active movement NRS score up to 4 h postoperatively. The incidence of postoperative nausea and vomiting (PONV) (*p* = 0.015, power = 70%), abdominal distension (*p* = 0.024, power = 64%), and muscular calf vein thrombosis (MCVT) (*p* = 0.033, power = 58%) was lower in the ESPB group than in the control group. Moreover, the occurrence of L-ESPB related adverse reactions was not found herein.

**Conclusion:**

US-guided L-ESPB reduces intraoperative and 24 h postoperative opioid consumption and improves patients' QoR-15 scores at 24 h postoperatively. L-ESPB can be safely and effectively utilized in lumbar UBE surgery.

**Trial registration:**

Chinese Clinical Trial Registry, ChiCTR2200061908, date of registration: 10/07/2022. Registry URL.

## Introduction

The unilateral bi-portal endoscopy (UBE) technique, which combines the advantages of microscopy and endoscopy, is a combination of open spine surgery and endoscopic spine surgery, and is increasingly being utilized for the treatment of degenerative lumbar spine diseases such as lumbar spinal stenosis and lumbar disc herniation [1, 2]. The lumbar UBE technique is a minimally invasive spine surgery (MISS), which is less disruptive to the local lumbar anatomy than open surgery; however, the technique also triggers the occurrence of intraoperative pain stimulation and postoperative pain adverse reactions, thereby affecting the patient's recovery process after surgery as well as the possibility of converting acute pain to chronic pain [3, 4]. Moreover, as in the case of open spine surgery, the postoperative pain management of minimally invasive UBE surgery should also be considered [5, 6].

The traditional method of analgesia after spinal surgery is based on opioid-based peripheral intravenous self-controlled analgesia [7]; however, the overdose application of opioids leads to adverse reactions [8], which limits the pain control efficacy. Previously, analgesia for lumbar spine surgery was limited to lumbar anesthesia, epidural anesthesia, and local infiltration anesthesia; however, their respective drawbacks limited their application. Although lumbar anesthesia and epidural can provide excellent analgesia, they can produce sympathetic or motor block, thereby affecting the judgement of postoperative neurological function [9]. Local infiltration anesthesia, the utilization of large amounts of drugs, and limited range, for superficial tissue pain control can be, for deep operation pain control is not effective [10].

The erector spinae plane block (ESPB), first proposed by Forero [11] in 2016, is categorized as a fascial plane blocks (FPB) method, which is a novel regional block technique that injects local anesthetics into the plane between the underside of the erector spinae muscle (ESM) and the transverse process (TP), thereby blocking the dorsal and ventral branches of the spinal nerves by diffusion. Because ESPB ultrasound (US)-guided anatomy is easily recognizable and far away from the pleura and crucial vascular nerves, it is simple and safe to operate, and is now gradually being utilized for analgesia in various surgeries [12–14], including shoulder, thoracic, and abdominal surgeries; lumbar spine surgeries; pilonidal sinus surgery; and orthopedic surgeries of the lower limbs. The efficacy and reliability of ESPB in posterior open lumbar spine surgery has been demonstrated [15]; however, few randomized controlled trials of lumbar ESPB (L-ESPB) in lumbar UBE surgery have been reported.

It is unclear whether L-ESPB for lumbar UBE surgery multi-modal analgesia is feasible. To examine the effectiveness and safety of L-ESPB for perioperative analgesia in lumbar UBE surgery, the current study evaluated and validated intraoperative and postoperative opioid consumption and postoperative quality of recovery-15 (QoR-15) scores as the main outcome indicators.

## Materials and methods

### Ethics

This study was approved by the Ethics Committee of Binzhou Medical University Hospital (Ethics approval number: 2022KT-09) and written informed consent was obtained from all subjects participating in the trial. The trial was registered prior to patient enrollment at the Chinese Clinical Trial Registry on July 10, 2022 (https://www.chictr.org.cn/showproj.html?proj=173379, ChiCTR2200061908, principal investigator: Dan Zhao).

### Study population

This prospective, randomized, controlled trial included adults aged 18–65 years (study period: between 10 July 2022 and 31 December 2022), who were to undergo UBE surgical decompression of the spinal canal or concomitant nucleus pulposus removal under general anesthesia. Exclusion criteria were as follows: (1) surgery involving ≥ 3 spinal segmental gaps; (2) American Society of Anesthesiologists (ASA) classification > Grade III; (3) surgery time > 3 h; (4) allergy to drugs in the study protocol; (5) history of previous surgery in the lumbar spine; (6) infection at the lumbar puncture site; (7) coagulation abnormality or ongoing anticoagulant medication; (8) history of analgesic medication abuse; (9) communication disorders; and (10) patients who did not accept to participate.

### Randomization and blinding

Prior to the commencement of the clinical trial, numbered groupings were completed independently by a non-participating researcher using computerized SPSS software (participants were consecutively enrolled and assigned the appropriate trial sequence numbers, namely 1 to 120), and participants were randomly allocated 1:1 to receive a single 0.25% ropivacaine L-ESPB on the operative side (the ESPB group) and a no-block group (the Control group). The grouping information was concealed in closed opaque envelopes affixed with serial numbers of consecutive trials, which were opened by only the chief anesthesiologist after the patient had entered the operating room. Throughout the clinical study, patients as well as dedicated postoperative follow-up staff were blinded to the grouping; however, the primary anesthetist and the primary surgeon were not blinded to it.

### US-guided unilateral single-shot L-ESPB

**Fig. 1:**
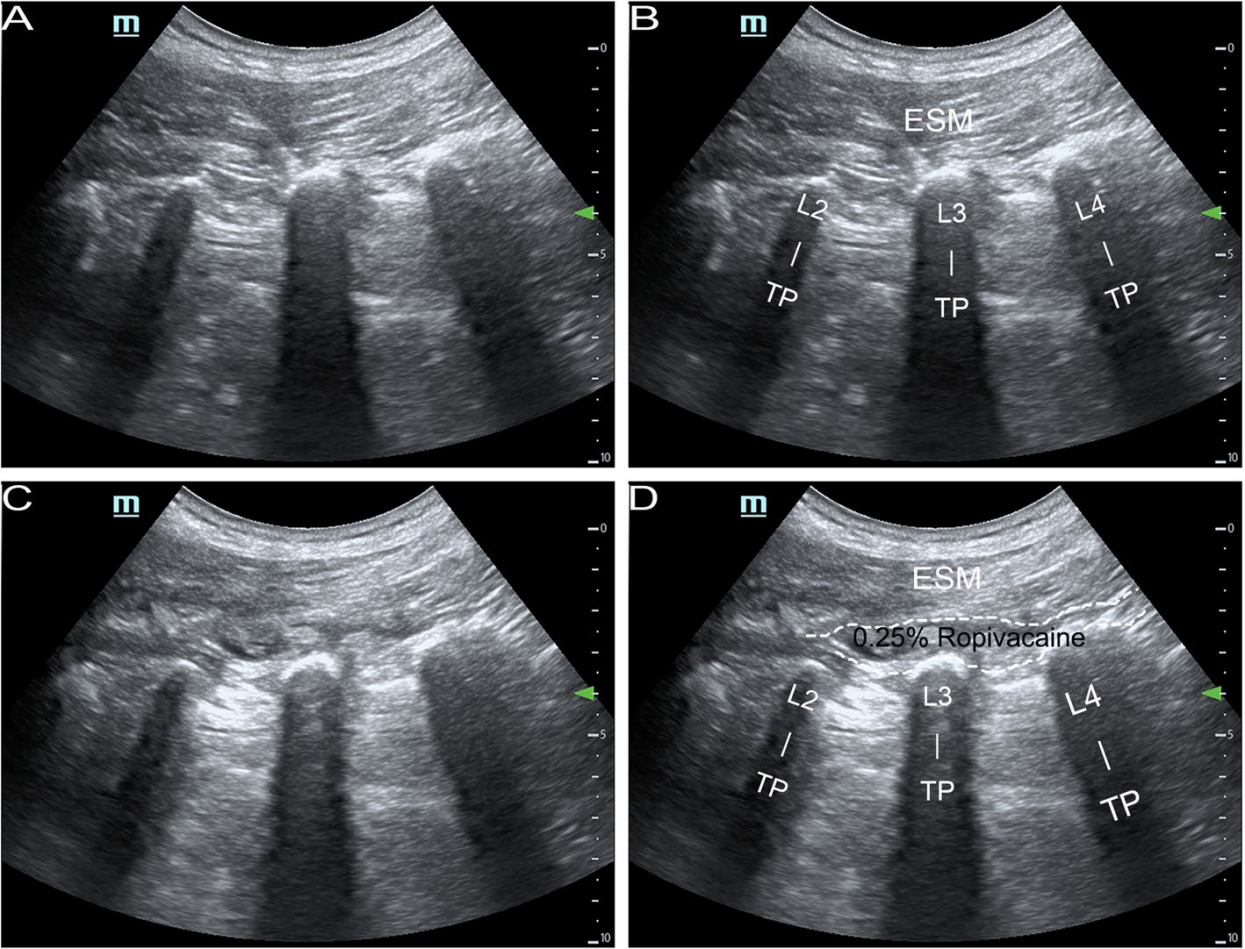
Images of ultrasound-guided lumbar erector spinae plane block (L-ESPB). **A** and **B** represent the anatomical structures associated with L-ESPB under ultrasound. **C** and **D** represent ultrasound-guided (in-plane) L-ESPB. The dashed area of D indicates the diffusion of 0.25% ropivacaine between the erector spinae muscle (ESM) and transverse processes. L2, L3 and L4 indicate the 2nd, 3rd and 4th lumbar vertebrae, respectively. TP indicates transverse processes

After induction of general anesthesia and completion of mechanical ventilation, the patient was positioned in the prone position on the surgical bed. The surgeon marked the UBE surgical incision using X-ray fluoroscopy with complete body positioning, and the anesthesiologist subsequently moved one more spinal segment cranially upwards from the position of the spinal segment to be operated on (UBE operative side) and performed an L-ESPB on its TP using the Tulgar approach [16] (the parasagittal approach in-plane), as shown in Fig. 1. Example: The patient was operated on in the L4/5 space, the UBE was performed on the right side of the midline, and the target site for ESPB was the right L3-TP. The patient was placed in the prone position, and a 2–5 MHz convex array ultrasound probe (C5-2 s, Mindray TE7, China) was placed parallel to the spine next to the right side of the spinal midline. Using X-ray fluoroscopy, the surgeon made a body marking and ultrasound identification of the iliac bone to identify the L3-TP in the upward direction, and subsequently moved the probe in the lateral direction (moving in a perpendicular direction to the spine), thereby identifying the articular process and the tip of the TP; moreover, the surgeon performed L-ESPB at the tip of the TP and the tip of the articular process, at the approximate midpoint of the two. Using the in-plane puncture technique (craniocaudal direction puncture), the puncture needle (Disposable Anesthetic Needles, AN-N 0.7*90, MEIXING, China) reached the region between the deep fascia of the ESM and the TP (the tip of the puncture needle arrived at the TP). After withdrawing no blood, the surgeon slowly injected 20 ml of 0.25% ropivacaine, with the diffusion of local anesthetic, and a certain thickness and length of the formation of a layer of liquid between the ESM and the TP could be observed.

### Perioperative management

The anesthetic and analgesic aspects of all patients participating in the clinical trial followed a standardized procedure. General anesthesia was induced by sequential intravenous midazolam 0.04 mg/kg, etomidate 0.3 mg/kg, fentanyl 4 mcg/kg, and cisatracurium 0.15 mg/kg. After completion of tracheal intubation and connection to a ventilator for assisted respiration, droperidol 1 mg and dexamethasone 5 mg were administered to prevent postoperative nausea and vomiting (PONV). Maintenance of anesthesia was effected using sevoflurane (inhalation concentration 1.7%-2.5%) and remifentanil (micro pump intravenous infusion 0.01–0.20 mcg·kg^−1^·min^−1^); according to the operation requirements, add cisatracurium 0.03 mg/kg (if the effect of inotropic relaxation is favorable, there is no need to add it in a timely manner) to maintain the 40–60 bispectral index (BIS) value. If the invasive blood pressure and heart rate increase by 20% or more or if controlled blood pressure reduction is required for surgery, remifentanil is administered 20–60 mcg once, and this procedure may be repeated. If additional cisatracurium was administered within 30 min before the end of the procedure, neostigmine 1 mg and atropine 0.5 mg were administered at the end of the procedure, thereby antagonizing the neuromuscular blockade. The endotracheal tube was removed when the patient was spontaneously breathing and conscious; subsequently, the surgeon determined the occurrence of spinal nerve root paralysis or motor block by examining the patient's dorsiflexion and plantarflexion of both feet and the movement of the lower limbs, and the patient was sent to the post-anesthetic care unit (PACU) to continue resuscitation. When the patient attained a modified Aldrete score of 10 in the PACU, he was then supervised and sent back to the ward.

### Postoperative pain management

All patients were given 0.5 mcg/kg fentanyl once approximately 30 min before the end of surgery. After the patient was awake for the removal of the endotracheal tube, a patient controlled intravenous analgesia (PCIA) pump was subsequently connected to the peripheral vein, which consisted of only diluted fentanyl. There was no initial additional dose, the continuous infusion rate was fentanyl 0.2 mcg·kg^−1^·h^−1^, and the additional PCIA Bolus was fentanyl 0.5 mcg/kg once, with a 30 min lock-in time. All patients were started on oral compound codeine phosphate and ibuprofen sustained release tablets (1 tablet containing ibuprofen 200 mg and codeine 13 mg, 1 tablet/q12h) for postoperative basal analgesia at 6 h postoperatively. The perioperative pain score was determined using the numeric rating scale (NRS, range 0–10), and when the NRS was ≥ 4 or there was a need for analgesia, fentanyl 0.5 mcg/kg was administered using a PCIA Bolus pump. However, for patients with NRS > 4 or unsatisfactory analgesia despite two consecutive Bolus additions of fentanyl by PCIA, tramadol 100 mg was intravenously administered at a slow rate on the ward as an intensive rescue analgesic measure. When patients exhibited PONV, intravenous droperidol 1 mg or intramuscular metoclopramide 10 mg was administered.

### Data collection and outcomes

First, the baseline characteristics of the patients and of the anesthesia and surgery were determined. Second, the main outcome indicators were identified: Total intraoperative remifentanil consumption (mcg) and consumption per kilogram of body weight per minute (mcg·kg^−1^·min^−1^); Consumption of fentanyl at 24 h and 48 h postoperatively; and QoR-15 scores at 12 h preoperatively (baseline), 24 h postoperatively, and 48 h postoperatively. Third, secondary outcome indicators were determined as follows: (1) Cisatracurium was utilized intraoperatively as well as the number of additions and the total number of additions. (2) Bolus numbers of the PCIA pump at 24 h and 48 h postoperatively, and time interval of the first postoperative Bolus (PCIA). (3) The change rate of a 10% increase in blood pressure or heart rate at the four intraoperative time points of skin incision, fascial dilation, clamping and biting of the periosteum, and retraction to release the nerve root. (4) NRS scores at PACU10min, PACU30min, and 2 h, 4 h, 8 h, 24 h, and 48 h of postoperative resting and active movement (change from supine to lateral position). (5) Postoperative opioid use-related adverse effects such as PONV, bloating, dizziness, and drowsiness, and L-ESPB related adverse effects (L-ESPB puncture-related hematoma and infection, and lower limb dyskinesia). (6) Incidence of muscular calf vein thrombosis (MCVT) at 48 h postoperatively.

### Statistical analysis

The results of the preliminary experiment with 10 cases each in the ESPB and control groups indicated that remifentanil consumption (mcg) was 256 ± 121.40 in the ESPB group and 510 ± 176.70 in the control group; fentanyl (mcg) at 24 h postoperatively was 388.50 ± 97.17 in the ESPB group and 454.10 ± 107.69 in the control group; and ESPB group QoR-15 scores at 24 h postoperatively were 119.70 ± 9.90 and 108.90 ± 10.98 in the control group. The sample size was estimated by intraoperative remifentanil consumption with α = 0.05 (2-sided), 90% power, and a 10% fallout rate, which was calculated to require 10 patients in each group. The sample size was estimated by QoR-15 scores at 24 h postoperatively with α = 0.05 (2-sided), 90% power, and a 10% fallout rate, which was calculated to require 24 patients in each group. The sample size was estimated by 24 h postoperative fentanyl consumption with α = 0.05 (2-sided), 90% power, and a 10% fallout rate, which was calculated to require 59 patients in each group. Ultimately, 60 patients were pre-included in this study in each group.

All statistical analyses were performed using IBM SPSS for Windows version 26.0 (IBM Corp., Armonk, NY, USA). Normality of data was assessed using the Shapiro–Wilk test. When the measurement data conformed to normal distribution, and when the variance was homogeneous: it was expressed as mean ± standard deviation (Mean ± SD), and two independent samples t-test were utilized for comparison between groups. When the measurement data did not meet the normal distribution or the variance was not uniform, it was expressed as the median and interquartile range [M(IQR)], and the two independent samples Mann–Whitney U test was utilized for comparison between groups. Count data were expressed as numbers (percentages) [n (%)], and comparisons between two groups were effected using Pearson's chi-squared test, Fisher's exact test, or the Mann–Whitney U test. A two-way repeated-measures analysis of variance was performed to assess the association between QoR-15 and NRS scores and time in the two groups. A 2-sided *p* < 0.05 was utilized to indicate a statistically significant difference.

## Results

**Fig. 2:**
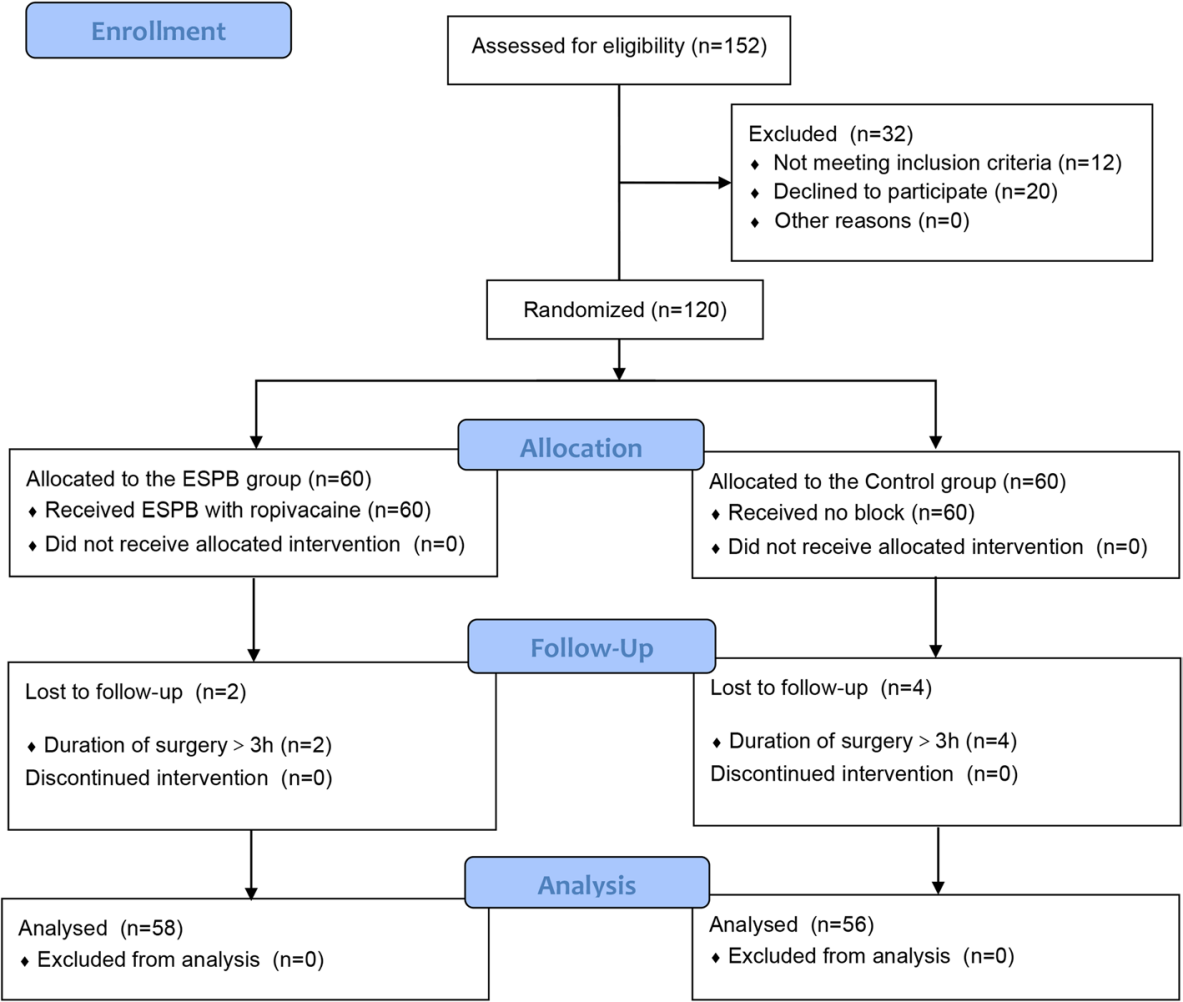
Consolidated standards of reporting trials (CONSORT) flow diagram. ESPB erector spinae plane block

The flowchart of the study is depicted in Fig. 2. A total of 120 patients were enrolled from 10 July 2022 to 31 December 2022. 152 patients were assessed for eligibility, 12 patients were excluded for taking large amounts of analgesic medication over a prolonged period of time, and 20 patients refused to be enrolled in the study. The final 120 patients were enrolled consecutively and randomly assigned in a ratio of 1:1 to the ESPB and the control groups. Two patients in the ESPB group and four patients in the control group were excluded for exhibiting a procedure time greater than 3 h. Finally, 58 patients in the ESPB group and 56 patients in the control group were included in the analysis. The power of intraoperative remifentanil consumption was 100%, the power of 24 h postoperative fentanyl consumption was 90%, and the power of 24 h postoperative QoR-15 score was 100%. No statistically significant differences were observed in patient baseline characteristics, surgical characteristics, and preoperative QoR-15 scores between the two groups, as illustrated in Table 1.

**Table 1:** Patient baseline characteristics and surgical data

Variable	ESPB (*n* = 58)	Control (*n* = 56)	*p* value
Age (years)	52[45–56]	53[48–59]	0.235^a^
Sex, n (%)			0.854^b^
Male	28(48.3)	28(50)	
Female	30(51.7)	28(50)	
Height (cm)	165[160–172]	165[160–172]	0.664^a^
Weight (kg)	70[63–80]	70[63–78]	0.337^a^
Weight for PCIA (kg)	70[60–80]	70[60–80]	0.363^a^
Weight difference (kg)	0[–1–4]	0[–1–3.5]	0.712^a^
BMI (kg/m^2^)	25.99[24.34–28.40]	24.75[23.40–27.27]	0.055^a^
ASA status, n (%)			0.824^a^
I	7(12.1)	6(10.7)	
II	46(79.3)	47(83.9)	
III	5(8.6)	3(5.4)	
Preoperative QoR-15 score	112[101–119]	108[100–117]	0.143^a^
Number of surgical intervertebral space, n (%)			0.916^b^
1	43(74.1)	42(75)	
2	15(25.9)	14(25)	
Surgical side, n (%)			0.170^b^
Left	22(37.9)	29(51.8)	
Right	16(27.6)	16(28.6)	
Bilateral	20(34.5)	11(19.6)	
Surgical procedure, n (%)			0.445^b^
Decompression	29(50)	24(42.9)	
Decompression and discectomy	29(50)	32(57.1)	
Duration of surgery (min)	85[70–108]	81[69–103]	0.939^a^
Duration of anesthesia (min)	117[96–143]	113[96–143]	0.872^a^
Anesthesia to surgery (min)	30[27–35]	30[25–36]	0.918^a^
L-ESPB to surgery (min)	22[19–27]	NA	NA
Length of PACU stay (min)	30[30–30]	30[30–30]	0.241^a^

Data are presented as median [IQR] or n (%)

*ESPB* Erector spinae plane block, PCIA Patient controlled intravenous analgesia, BMI Body mass index, *ASA* American Society of Anesthesiologists, *QoR-15* Quality of recovery-15, *Weight for PCIA* To facilitate the calculation of fentanyl dosage, the corrected body weight for the configuration of the PCIA pump was obtained in this study by rounding the single-digit value of the initial body weight (for example, body weight of 74 kg was transformed to 70 kg, and body weight of 76 kg was transformed to 80 kg)

^a^Mann-Whitney U test

^b^Pearson’s chi-squared test

### Primary outcomes

Total intraoperative remifentanil consumption (mcg, *p* < 0.001) and remifentanil consumption (mcg·kg^−1^·min^−1^, *p* < 0.001) were significantly lower in the ESPB group than in the control group (Table 2). Fentanyl consumption at 24 h (*p* = 0.001) postoperatively was significantly lower in the ESPB group than in the control group; however, fentanyl consumption at 24-48 h (*p* = 0.423) postoperatively and 48 h (*p* = 0.051) postoperatively was similar in both groups (Table 2). The two-way repeated measures anova indicated that there was a significant interaction between group and time for QoR-15 (*p* < 0.001). QoR-15 scores were significantly higher in the ESPB group than in the control group at 24 h (*p* < 0.001) and 48 h (*p* < 0.001) postoperatively (Table 2).

**Table 2:** The primary outcomes and some other perioperative indicators

Perioperative indicators	ESPB (*n* = 58)	Control (*n* = 56)	Median difference [95%confident interval]	*p* value
Postoperative 24 h QoR-15 score	123[119–127]	113[107–116]	11[8 to 14]	< 0.001^a^
Postoperative 48 h QoR-15 score	129[127–132]	125[123–127]	4[3 to 5]	< 0.001^a^
Remifentanil consumption (mcg)	390[240–520]	620[500–820]	–280[–360 to –200]	< 0.001^a^
Remifentanil consumption (mcg/kg/min)	0.043[0.033–0.055]	0.080[0.064–0.099]	–0.037[–0.044 to –0.029]	< 0.001^a^
Cisatracurium consumption (mg)	10.5[9.69–12]	10.8[9.56–12]	0[–0.75 to 0.50]	0.784^a^
Additional intraoperative dose (mg)	0[0–0]	0[0–1]	0[0 to 0]	0.004^a^
Number of intraoperative additions	0[0–0]	0[0–1]	0[0 to 0]	0.006^a^
Fentanyl consumption for PCIA (mcg)				
0-24 h fentanyl consumption (mcg)	396[336–491]	476[408–596]	–80[–128 to –32]	0.001^a^
24 h-48 h fentanyl consumption (mcg)	406[348–476]	406[348–424]	0[–18 to 46]	0.423^a^
0-48 h fentanyl consumption (mcg)	808[707–1001]	882[756–1028]	–70[–140 to 0]	0.051^a^
PCIA for patients				
Time interval for the first bolus (h)	9[6.75–28.5]	4[2–5]	6[4 to 14]	< 0.001^a^
Number of postoperative 24 h bolus	2[0–4]	4[4–6]	–3[–4 to –2]	< 0.001^a^
Number of postoperative 24-48 h bolus	1[1, 2]	1[1, 2]	0[0 to 0]	0.925^a^
Number of postoperative 48 h bolus	3[1–5]	6[5–8]	–3[–4 to –2]	< 0.001^a^

Data are presented as median [IQR]

*ESPB* Erector spinae plane block, *QoR-15* Quality of recovery-15, *PCIA* Patient controlled intravenous analgesia

^a^Mann-Whitney U test

### Secondary outcomes

The two-way repeated measures anova indicated that there was a significant interaction between group and time for pain score at rest (*p* < 0.001). The ESPB group significantly reduced resting NRS at the five postoperative time points of PACU10min (*p* < 0.001), PACU30min (*p* < 0.001), 2 h (*p* < 0.001), 4 h (*p* < 0.001), and 8 h (*p* = 0.029) compared to the control group; however, resting NRS were similar at 24 h (*p* = 0.397) and at 48 h (*p* = 0.541) postoperatively. Comparison of the area under the curve (AUC, estimated by the trapezoidal method) for NRS-time between the two groups revealed that the ESPB group reduced only the AUC of postoperative PACU10min to 8 h (*p* < 0.001), (Table 3). Two-way repeated measures anova indicated that there was a significant interaction between group and time for pain score on active movement (*p* < 0.001). The ESPB group significantly reduced active movement NRS at postoperative PACU10min (*p* < 0.001), PACU30min (*p* < 0.001), 2 h (*p* < 0.001), and 4 h (*p* < 0.001) compared to the control group; however, active movement NRS were similar at all three postoperative times, namely 8 h (*p* = 0.977), 24 h (*p* = 0.680), and 48 h (*p* = 0.124). Comparison of the AUC for NRS-time between the two groups indicted that the ESPB group similarly reduced only the AUC of postoperative PACU10min to 8 h (*p* < 0.001), (Table 3). The time interval between the first Bolus of the PCIA pump was longer in the ESPB group than in the control group (*p* < 0.001). The Bolus numbers of the PCIA pump in the 24 h postoperative period was significantly lower in the ESPB group than in the control group (*p* < 0.001), whereas the Bolus numbers of the PCIA pump in the 24 h to 48 h postoperative period was similar in both groups (*p* = 0.925) (Table 2).

**Table 3:** Postoperative pain score and the area under the curve for NRS-time

Time/time interval	ESPB (*n* = 58)	Control (*n* = 56)	Median difference [95%confident interval]	*p* value
NRS at rest				
PACU_10min_	0[0–0]	1[0–2]	–1[–1 to –1]	< 0.001^a^
PACU_30min_	0[0–0]	2[1, 2]	–2[–2 to –1]	< 0.001^a^
2 h	1[0–1]	2[2, 3]	–1[–2 to –1]	< 0.001^a^
4 h	1[1, 2]	2[2, 3]	–1[–1 to –1]	< 0.001^a^
8 h	2[1, 2]	2[2, 3]	0[–1 to 0]	0.029^a^
24 h	1[1, 2]	1[1, 2]	0[0 to 0]	0.397^a^
48 h	1[1, 2]	1[1–1]	0[0 to 0]	0.541^a^
AUC of PACU_10min_-8 h	9.33[5–13.92]	17.45[13–21.70]	–7.58[–9.58 to –5.58]	< 0.001^a^
AUC of 8 h-24 h	24[16–32]	32[24–40]	0[–8 to 0]	0.377^a^
AUC of 24 h-48 h	30[24–36]	24[24–36]	0[0 to 0]	0.388^a^
AUC of 8 h-48 h	56[40–76]	56[48–76]	0[–8 to 8]	0.929^a^
AUC of PACU_10min_-48 h	63.50[51–90.17]	74.46[62.35–93.33]	–6.87[–17.33 to 1.76]	0.092^a^
NRS on active movement				
PACU_10min_	0[0–0]	2[1–3]	–2[–2 to –1]	< 0.001^a^
PACU_30min_	0[0–1.25]	3[2, 3]	–2[–2 to –2]	< 0.001^a^
2 h	2[1–3]	3[2–5]	–2[–2 to –1]	< 0.001^a^
4 h	2[2–3.25]	4[2.25–5]	–1[–2 to –1]	< 0.001^a^
8 h	4[3–5]	4[3–5]	0[–1 to 1]	0.977^a^
24 h	3[2–4]	3[2, 3]	0[0 to 1]	0.680^a^
48 h	3[2, 3]	2[2, 3]	0[0 to 1]	0.124^a^
AUC of PACU_10min_-8 h	19.46[13.75–24.52]	29.37[21.56–35.29]	–9.25[–12.83 to –5.83]	< 0.001^a^
AUC of 8 h-24 h	52[40–64]	56[40–64]	0[–8 to 8]	0.909^a^
AUC of 24 h-48 h	60[57–84]	60[48–84]	0[0 to 12]	0.276^a^
AUC of 8 h-48 h	120[96–145]	114[92–143]	4[–8 to 20]	0.538^a^
AUC of PACU_10min_-48 h	136.29[107.17–170.75]	146.83[110.43–176.35]	–4.87[–21.75 to 11.25]	0.518^a^

Data are presented as median [IQR]

*ESPB* Erector spinae plane block, *NRS* Numeric rating scale (range 0–10), *PACU* Post-anesthetic care unit, *AUC* Area under the curve

^a^Mann-Whitney U test

The total intraoperative cisatracurium consumption was similar in both groups (*p* = 0.784); however, the number of intraoperative cisatracurium additions (*p* = 0.004) and the amount of additions (*p* = 0.006) were significantly lower in the ESPB group compared with the control group (Table 2). At the four time points of intraoperative skinning (*p* = 0.001), fascial dilatation (*p* < 0.001), clamping of the periosteum (*p* < 0.001), and distraction and release of the nerve roots (*p* < 0.001) in the UBE procedure, there was a significantly lower rate of positive change (a 10% elevation in blood pressure and/or heart rate) in the ESPB group compared with the control group ( Table 4).

**Table 4:** Other results on response rates during the study period

Other results	ESPB (*n* = 58)	Control (*n* = 56)	*p* value
Four invasive maneuvers during lumbar UBE surgery:			
Cut the skin, n (%)	3(5.2)	16(28.6)	0.001^a^
Dilation of myofascial, n (%)	8(13.8)	48(85.7)	< 0.001^a^
Pincer bite periosteum, n (%)	5(8.6)	22(39.3)	< 0.001^a^
Pulling and releasing nerve roots, n (%)	26(44.8)	54(96.4)	< 0.001^a^
Opioid-related adverse events			
Occurrence of PONV, n (%)	10(17.2)	21(37.5)	0.015^a^
Occurrence of distended abdomen, n (%)	6(10.3)	15(26.8)	0.024^a^
Occurrence of dizziness, n (%)	1(1.7)	2(3.6)	0.615^b^
Occurrence of drowsiness, n (%)	3(5.2)	1(1.8)	0.619^b^
L-ESPB related adverse event, n (%)	0(0)	NA	NA
MCVT within 48 h after surgery, n (%)	3(5.2)	10(17.9)	0.033^a^

Data are presented as n (%)

*ESPB* Erector spinae plane block, *UBE* Unilateral bi-portal endoscopic, *PONV* Postoperative nausea and vomiting, *MCVT* Muscular calf vein thrombosis

^a^Pearson’s chi-squared test

^b^Fisher's exact test

Tramadol was not utilized as intensive rescue analgesia in either group. For postoperative opioid-related adverse reactions, the incidences of PONV (*p* = 0.015, power = 70%) and abdominal distension (*p* = 0.024, power = 64%) was lower in the ESPB group than in the control group, and the incidence of other adverse reactions was similar in both groups. Postoperative incidence of MCVT was lower in the ESPB group than in the control group (*p* = 0.033, power = 58%). The occurrence of L-ESPB related adverse reactions was not identified herein (Table 4).

## Discussion

This randomized controlled clinical trial demonstrated that US-guided unilateral single-shot L-ESPB reduced intraoperative and 24 h postoperative opioid consumption, increased postoperative QoR-15 scores in patients undergoing lumbar UBE surgery, improved the resting NRS score up to 8 h postoperatively and active movement NRS score up to 4 h postoperatively, significantly lengthened the interval between rescue analgesia, and reduced postoperative opioid-related side effects. These results indicate that L-ESPB can still provide effective intraoperative and postoperative analgesia despite the flushing effect of large amounts of intraoperative irrigation fluid in lumbar UBE surgery.

The ESPB mechanism of action is clearly identified in the literature [11, 17]. However, the mechanism of action of L-ESPB is quite controversial. Most cadaveric studies have revealed that L-ESPB effectively blocks the dorsal branch of the spinal nerve, whereas the block of the ventral branch pertaining to the spinal nerve is not definitive [18–24]. Regional anesthesia methods for lumbar surgery should cover the innervation of the relevant vertebrae and paravertebral muscles, and include the dorsal branch of the spinal nerve at that level [25, 26]. Blockade of the dorsal branch of the spinal nerve by local anesthetics may be the main L-ESPB mechanism, and the surgical site of UBE surgery, which is predominantly innervated by the dorsal branch of the spinal nerve, is just right for posterior lumbar spine surgery [18–26], even if there is no paravertebral diffusion to block the ventral branch of the spinal nerve. The systemic effect of local anesthetics injected into the fascial planes is one of the most popular proposed mechanism for action for L-ESPB; this effect may have influenced the UBE surgeries [27]. Numerous studies have demonstrated the effective analgesic effect of ESPB in posterior open lumbar spine surgery [15]. The myofascial structure of the lumbar segment is more complex and variable than that of the thoracic segment, and L-ESPB diffusion is relatively limited [21]. Anatomical studies have revealed that a median of 5 ml of local anesthetic is required to cover one vertebrae at the lumbar level [28], and because we performed L-ESPB in the last vertebral plane at the operative stage, the volume was set at 20 ml [24, 28]. Because the UBE surgical incision was mainly concentrated on one side and bilateral spinal decompression was feasible for the operation of one side of the UBE [29], we chose unilateral L-ESPB.

In a recent retrospective study conducted by Tae Hoon Kang et al. [25], it was found that L-ESPB was not different from spinal anesthesia (SA) but superior to general anesthesia (GA) for postoperative pain management; the study demonstrated that L-ESPB combined with sedation is a viable anesthetic option for lumbar decompression surgery in UBE, thereby more effectively investigating the effectiveness of L-ESPB as a block in posterior spinal endoscopic procedures for UBE. Herein, we observed whether the patients' blood pressure and or heart rate increased by 10% of the baseline at the time of the four highly traumatic and painful stress-responsive surgical operations, namely skinning, myofascial dilation, clamping and biting of the periosteum, and retraction and release of the nerve heel; moreover, we found that the ESPB group exhibited a lower rate of positive reaction than the control group, and that the lower intraoperative remifentanil consumption in the ESPB group compared with the control group indirectly demonstrated the blocking effect of L-ESPB on the dorsal branch of the spinal nerve.

This study indicated that L-ESPB improved resting NRS up to 8 h postoperatively and active movement NRS up to 4 h postoperatively. Compared with the previous ESPB utilized for open spine surgery [30, 31], pre-operative US-guided L-ESPB improved postoperative NRS for a shorter duration, which we interpreted as, firstly, the effect of the large volume of irrigation fluid during UBE surgery, which continuously flushes the surgical site and dilutes the concentration and total dose of local anesthetic drugs, attenuating the analgesic duration of L-ESPB. Second, we used a unilateral L-ESPB, missing the overlay of local anesthetic drug diffusion in the other side of the L-ESPB. Third, we used a lower concentration of local anesthetic drug to perform the L-ESPB. Fourth, despite having a median of 22 min, the time interval between the L-ESPB and the beginning of the procedure was not always more than 20 min, and the current study did not measure the extent of sensory block. Although there are individual differences in the existing research, it is generally accepted that the minimal clinically crucial difference (MCID) in postoperative pain scores after lumbar spine surgery is 1.2 points [32]. Herein, only the active movement NRS within 2 h postoperative period and the resting NRS at one moment in the PACU30min was improved more than the MCID. However, the AUC (NRS-time) for the postoperative PACU10min-8 h period and the consumption of fentanyl at 24 h postoperatively were significantly lower in the ESPB group than in the control group. These valuable findings indirectly indicate the effective dorsal spinal nerve branch blocking effect of L-ESPB and its local analgesic effect.

Yörükoğlu et al. [33] conducted a study wherein unilateral ESPB (20 mL of 0.25% bupivacaine) was administered for postoperative analgesia in patients undergoing single-segment microdiscectomy of the lumbar intervertebral discs. The study's findings showed that ESPB decreased the amount of morphine that patients took during the 24-h postoperative period (11.3 ± 9.5 mg vs. 27 ± 16.7 mg). However, no statistically significant difference was observed in the NRS scores of the two groups following the procedure. In a research study conducted by Amarjeet Kumar et al. [34], the effectiveness of ESPB was compared to Modified thoracolumbar Interfascial Plane Block (mTLIP) for managing postoperative pain after lumbar spine surgery. The findings indicated that ESPB was more successful in reducing 24-h postoperative fentanyl usage (89.9 ± 65.3 mcg vs. 150.3 ± 120.9 mcg) compared to mTLIP. In the current study, the consumption of postoperative opioids was significantly higher than in studies in which ESPB was applied to other spinal surgical procedures [15, 33, 34], and our explanation is because of several points. Firstly, in this study, no injections (paracetamol injection, acetaminophen and flurbiprofen axetil injection, etc.) of Nonsteroidal Antiinflammatory Drugs (NSAIDs) for hyperalgesia or other types of opioids (tramadol, pethidine and hydromorphone, etc.) for additional analgesia were used in this study. Secondly, in this study, compound codeine phosphate and ibuprofen sustained release tablets, which were used as basic analgesia, were administered late (oral administration started after 6 h postoperatively) and in small dosages (The instructions are for 2 tablets/12 h, in this study it was 1 tablet/12 h. 1 tablet containing ibuprofen 200 mg and codeine 13 mg.). Thirdly, postoperative pain after lumbar UBE is mainly known as acute pain in the early postoperative period, while postoperative analgesia in this study was mainly fentanyl-based, with no other opioids additionally used as rescue analgesia. These three reasons led to the high consumption of postoperative fentanyl in both groups in this study. The study showed that the use of L-ESPB resulted in a significant decrease in fentanyl consumption in the 24-h postoperative period compared to the control group, indicating the effectiveness of L-ESPB in reducing postoperative pain in lumbar UBE. In a retrospective clinical study by Hironobu Ueshima et al. [35], bilateral ESPB (each side, 20 ml of 0.375% bupivacaine) was used for analgesia after lumbar microendoscopy, and all patients received PCIA after surgery that omprised 0.5 mcg·kg^−1^·h^−1^ continuous fentanyl administration and 0.5 mcg/kg fentanyl bolus infusion when requested by the patients. ESPB was found to reduce the number of postoperative fentanyl bolus infusions (bolus), and its postoperative fentanyl consumption and the number of fentanyl bolus infusions were similar to the results of this study.

Subbiah et al. [9] and Goel et al. [36], revealed that ESPB was able to reduce the amount of intraoperative muscarinic medication in open thoracolumbar spine surgery, interpreted as relieving tension in the ESM. The current study similarly found a significant reduction in the number of intraoperative myosin additions and the amount of additions in the ESPB group; however, there was no difference in the total amount of intraoperative myosin between the two groups. The preceding observation may be rationalized using two explanations: (1) this study was not blinded to the surgeon and the primary anesthesiologist, and there may be a bias in the outcome metrics observed intraoperatively; (2) although L-ESPB was effective in reducing the additional need for muscular relaxation in lumbar UBE surgery, overall, lumbar UBE surgery exhibited lower muscular relaxation requirements compared to open spine surgery.

The QoR-15 score scale has been utilized in assessing the recovery of patients after lumbar spine surgery [31, 37]. Herein, the statistical results indicated that L-ESPB improved the QoR-15 scores of posterior lumbar UBE surgery at 24 h and 48 h postoperatively, and promotes the early recovery of patients in the postoperative period. However, the MCID for the QoR-15 score is 8 points [38]; therefore, this study only significantly improved the QoR-15 score at 24 h postoperatively, whereas there was only a statistically significant difference between the two groups on the QoR-15 score at 48 h postoperatively, with no clinically meaningful improvement. PONV is a key driver of patient satisfaction, recovery, and readiness for discharge [39]. The reduced incidence of postoperative PONV and bloating in the ESPB group was interpreted to be related to the perioperative utilization of fewer opioids in patients in the ESPB group. Patients were usually requested by the surgeon to stay out of bed for 48 h postoperatively. Therefore, the time pertaining to the first time out of bed was not recorded; however, the occurrence of MCVT was utilized to react to the patient's lower limb activity. In the 48 h postoperative period, the lower MCVT incidence in the ESPB-group patients was explained as follows: the effective preemptive analgesic effect of L-ESPB was related to the reduction of acute intraoperative and postoperative pain stress and favorable active lower limb muscle movement in the postoperative period. It was demonstrated that L-ESPB could reduce peripheral and central nervous system sensitization occasioned by perioperative harmful stimuli in patients with UBE [40, 41]; cut off the pain chain; increase the pain threshold, and reduce the postoperative pain intensity; and reduce analgesic drug requirements and drug-related adverse effects. The higher postoperative QoR-15 scores in the ESPB group benefited from effective intraoperative and postoperative analgesia, and were associated with a lower incidence of postoperative PONV and abdominal distension, as well as less postoperative MCVT incidence.

Limitations: First, this study was a single-blind randomized controlled clinical trial, and although we blinded patients and followers, we did not blind the primary anesthesiologist or the primary surgeon, which would have biased the results. Second, a 20 ml volume was utilized to implement L-ESPB, and although it appeared to be sufficient for blocking the dorsal branches of the spinal nerves in the target area, the volume was not calculated based on kilograms of body weight. Third, L-ESPB was performed after induction of general anesthesia in patients without assessing the extent of sensory block, and the control group was not given a sham block with an equivalent volume of saline because we inferred it was ethically inappropriate. Fourth, we included patients with lumbar disc herniation and lumbar spinal stenosis (the most common conditions for which UBE surgery is clinically performed), and did not qualify the UBE surgery for one condition separately, which could have biased the results.

In conclusion, US-guided unilateral single-shot L-ESPB reduces intraoperative and 24 h postoperative opioid consumption and improves patients' QoR-15 scores at 24 h postoperatively. Thus, L-ESPB can be safely and effectively utilized in lumbar UBE surgery.

## Data Availability

The datasets generated during and/or analyzed during the current study are available from the corresponding author on reasonable request.
